# Variation in Practice of the Diagnostic Workup of Asymptomatic Patients Diagnosed with Invasive Breast Cancer

**DOI:** 10.3389/fonc.2016.00056

**Published:** 2016-03-11

**Authors:** Anees B. Chagpar, Gildy V. Babiera, Jose Aguirre, Kelly K. Hunt, Tyler Hughes

**Affiliations:** ^1^Department of Surgery, Yale University, New Haven, CT, USA; ^2^Department of Surgery, University of Texas MD Anderson Cancer Center, Houston, TX, USA; ^3^Department of Surgery, Hospital de los Valles, Quito, Ecuador; ^4^Department of Surgery, McPherson Hospital, McPherson, KS, USA

**Keywords:** variation, breast cancer, workup, tests, guideline adherence

## Abstract

**Introduction:**

Breast cancer is frequently diagnosed, yet variation remains in terms of practice patterns in presurgical workup. We sought to determine factors associated with this variation.

**Methods:**

An anonymous web-based survey was distributed to surgeons regarding their practices. Statistical analyses were conducted using SPSS.

**Results:**

A total of 253 surgeons responded to the survey. 17.0% were in academic practice, 37.5% were hospital employed, and 41.5% were in private practice. 53.3% claimed that >50% of their practice was breast related. Surgeons were asked how often they would use various tests in the workup of an *otherwise healthy asymptomatic patients, presenting with a non-palpable mammographic abnormality and a core needle biopsy showing invasive breast cancer*. 23.5% stated that they *always* would obtain a breast ultrasound, 17.2% stated that they *never* would. 12.8% stated that they *never* order a breast MRI; 4.1% *always* would. Workup of patients did not vary significantly based on number of years in practice nor practice setting. However, those whose practice was >50% breast were more likely to state that they would *always* order a breast ultrasound (32.5 vs. 12.9%, *p* < 0.001), and less likely to state that they would *never* order a breast MRI (3.4 vs. 25.8%, *p* < 0.001). However, the proportions of surgeons who would *always* order a breast MRI were similar in the two groups (3.4 and 3.2%, respectively).

**Conclusion:**

These data highlight the lack of uniformity in the workup of asymptomatic patients presenting with non-palpable breast cancers, pointing to potential areas for improving value by minimizing variability.

## Introduction

Breast cancer is the leading malignancy affecting women, with over 232,000 people being diagnosed with this disease annually in the United States (1). Given the ubiquity of screening mammography, particularly in more developed parts of the world, many women will present with non-palpable early stage disease. Faced with a plethora of potential adjunct imaging modalities, there is significant variability in the workup of these patients. Evidence for the cost-effectiveness and/or incremental benefit of these additional imaging techniques is limited. While clinical practice will vary based on individual patient characteristics, the goal of this study was to determine, given a hypothetical scenario, how often surgeons felt they would order various tests. While guidelines advocate for or against some of these techniques in all circumstances, they are far less prescriptive for others. We, therefore, sought to determine the variation in practices among surgeons in their preoperative workup of otherwise healthy asymptomatic patients presenting with non-palpable early stage breast cancer.

## Materials and Methods

An anonymous web-based survey was distributed to surgeons via the American College of Surgeons Communities. The Communities is an online platform, with a variety of discussion forums geared toward individual surgeons’ interests and demographics. Our survey was posted in the General, Breast Surgery and International forums. We asked surgeons to “*tell us how [they] work up patients who present to [them] with a newly diagnosed breast cancer. For otherwise healthy asymptomatic patients, presenting with a non-palpable mammographic abnormality and a core biopsy showing invasive breast cancer, [surgeons were asked] how often [they] perform each of [a prespecified list of] tests as part of [their] presurgical work-up*.” For each option, they could choose from the following options: *never, infrequently (*<*25%), sometimes (25–75%), often (*>*75%), or always*. No identifiable personal information was collected. Given the nature of the survey, it was deemed exempt by the Human Investigations Committee of Yale University.

Non-parametric bivariate statistical analyses using Fisher’s exact and likelihood ratio tests for categorical variables were conducted using IBM SPSS Statistics (Version 21). As this was a survey that was deemed to be hypothesis generating rather than hypothesis testing, a power analysis and/or sample size calculation was not indicated. The survey was posted on an online platform and, therefore, it is impossible to determine the denominator for a potential response rate.

## Results

Two hundred fifty three surgeons from eight countries, primarily in North America, responded to the survey. The majority (*n* = 234) came from the United States. Their demographic and practice characteristics are shown in Table 1. Surgeons were asked how frequently they would order each of a series tests in the setting of an otherwise healthy asymptomatic woman who presented with a non-palpable mammographic abnormality and a core needle biopsy result of invasive ductal carcinoma.

**Table 1:** **Demographic and practice characteristics of respondents**.

Factor	Number of respondents (%)
Age (years)^a^	
30–40	38 (15.0)
41–50	54 (21.3)
51–60	76 (30.0)
61–70	54 (21.3)
>70	16 (6.3)
Years in practice^b^	
<5	26 (10.3)
5–10	31 (12.3)
11–20	53 (20.9)
21–30	78 (30.8)
>30	52 (20.6)
Proportion of practice breast related (%)^c^	
<10	25 (9.9)
10–25	60 (23.7)
26–50	22 (8.7)
51–75	27 (10.7)
76–99	34 (13.4)
100	74 (29.2)
Practice setting^d^	
Private practice	105 (41.5)
Hospital employed	95 (37.5)
Academic	43 (17.0)

*^a^Age group not specified by 15 (5.9%) respondents*.

*^b^Years in practice not specified by 13 (5.1%) respondents*.

*^c^Proportion of practice that is breast related not specified by 11 (4.3%) respondents*.

*^d^Practice setting not specified by 10 (4.0%) respondents*.

### Additional Breast Imaging

**Figure 1:**
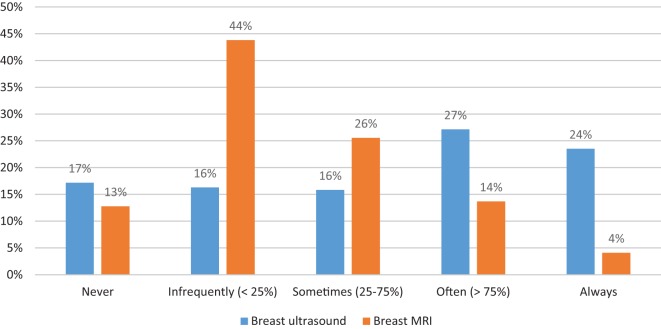
**Distribution of additional breast imaging**. Proportion of respondents reporting their likelihood of ordering either breast ultrasound (blue bars) or breast MRI (orange bars).

In terms of additional breast imaging, there was significant variation in practice. 17.2% of respondents stated that they would “*never*” order a breast ultrasound in this circumstance, while 23.5% stated that they “*always*” would. The distribution of responses were fairly evenly distributed between the five possible responses of *never, infrequently (*<*25%), sometimes (25–75%), often (*>*75%), and always*. In terms of breast MRI, nearly three times as many respondents reported that they would never order this test in this circumstance (12.8%) as those who stated that they always would (4.1%). These distributions are shown in Figure 1. There was a significant correlation between the use of breast ultrasound and breast MRI, with 40.5% of the 37 surgeons who stated that they “never” order breast ultrasound also noting that they would never use breast MRI, and 28.6% of the seven surgeons who stated that they would “always” order breast MRI also stating that they would always order a breast ultrasound (*p* < 0.001).

There was no correlation between surgeon age, number of years in practice, type of practice setting and geographic location, and the propensity to order additional breast imaging (Table 2). The only demographic or practice variable associated with the use of breast ultrasound and/or MRI was the proportion of practice that was breast related. Respondents whose practice was >50% breast related were significantly more likely to report that they would “*always*” order a breast ultrasound (32.5 vs. 12.9%, *p* < 0.001). While the proportion of those who stated that they would “*always*” order a breast MRI was not remarkably different between those with greater than vs. less than or equal to 50% breast related (3.4 vs. 3.2%, respectively), those with ≤50% breast-related practices were significantly more likely to “*never*” order a breast MRI in this circumstance (25.8 vs. 3.4%, *p* < 0.001).

**Table 2:** **Factors correlating with the use of further breast imaging**.

Factor	Breast US		Breast MRI	
	No. (%) reporting “always”	*p*-value	No. (%) reporting “always”	*p*-value
Age (years)		0.200		0.581
30–40	10 (29.4)		2 (5.7)	
41–50	12 (23.5)		1 (2.1)	
51–60	12 (18.2)		4 (5.8)	
61–70	13 (27.7)		1 (2.1)	
>70	4 (26.7)		1 (6.7)	
Years in practice		0.590		0.326
<5	7 (30.4)		2 (8.3)	
5–10	9 (33.3)		0 (0)	
11–20	10 (21.7)		1 (2.2)	
21–30	11 (16.4)		5 (7.5)	
>30	13 (27.1)		1 (2.2)	
% Practice breast related		0.005		<0.001
<10	2 (9.1)		0 (0)	
10–25	7 (13.7)		2 (3.8)	
26–50	3 (15.0)		1 (5.0)	
51–75	6 (27.3)		1 (4.3)	
76–99	9 (32.1)		0 (0)	
100	24 (34.3)		3 (4.3)	
Practice setting		0.440		0.194
Private practice	20 (21.7)		4 (4.3)	
Hospital employed	19 (21.8)		4 (4.7)	
Academic	11 (29.7)		0 (0)	

### Metastatic Workup

**Figure 2:**
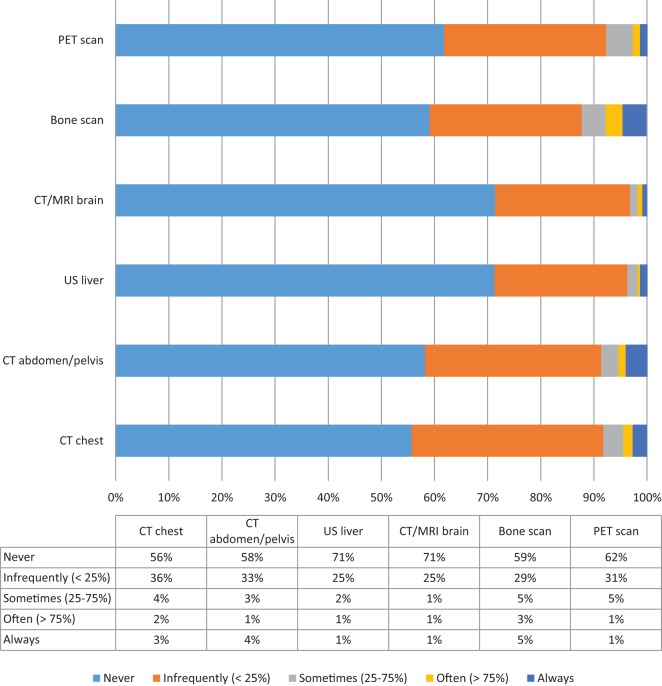
**Distribution of tests typically used for the evaluation of distant metastatic disease**. Proportion of respondents reporting their likelihood of ordering CT scan chest, CT scan abdomen/pelvis, Ultrasound liver, CT/MRI brain, Bone scan, PET scan.

In terms of tests that may be ordered as part of a metastatic workup, the majority of respondents stated that they would “*never*” order a CT chest, CT abdomen/pelvis, ultrasound of the liver, bone scan, CT/MRI brain, or PET scan in asymptomatic patients presenting with non-palpable mammographically detected (presumed early stage) disease. The distribution of responses for each of these tests is shown in Figure 2.

There were neither demographic nor practice-related variables that significantly correlated with the use of additional imaging to rule out metastatic disease among surgeons. Interestingly, those who practiced outside of the United States were significantly more likely to report that they would “*always*” obtain an ultrasound of the liver (13.3 vs. 0.5%, *p* = 0.001), and a bone scan (26.7 vs. 2.5%, *p* = 0.004) than their American counterparts. Rates of ordering CT or PET scans did not vary significantly based on geographic location.

Not surprisingly, surgeons who stated that they only obtain a metastatic workup in patients presenting with clinically stage III disease were less likely to report “*always*” obtaining a CT chest (0 vs. 5.9%, *p* = 0.008), CT abdomen/pelvis (0 vs. 7.9%, *p* = 0.010), or a bone scan (0 vs. 7.9%, *p* = 0.004) in the situation of an asymptomatic patient presenting with non-palpable, mammographically detected cancer.

### Chest X-Rays and “Routine” Blood Work

Surgeons’ use of “routine” blood work and chest X-rays (CXRs) as part of the preoperative workup for these patients was also elicited. We did not expressly ask surgeons whether these tests were being done to rule out metastatic disease or whether these were being done as part of their perioperative management. Blood work, such as complete blood counts (CBC), comprehensive metabolic panel (CMP)/electrolytes, and liver function tests (LFTs) were frequently performed, with 56.6, 53.3, and 48.9% of surgeons stating that they would “*always*” do each of these tests, respectively. The use of CXR was much more variable, with 29.9% of respondents stating that they would “*never*” order a CXR in this setting and 33.9% stating that they “*always*” would.

There were few characteristics that were associated with the use of these tests. Respondents whose practices were entirely breast surgery were more likely to report “*never*” ordering CXR (44.1 vs. 23.3%, *p* = 0.005), CBC (18.8 vs. 6.0%, *p* = 0.020), CMP/electrolytes (24.6 vs. 6.6%, *p* = 0.003), or LFTs (30.4 vs. 11.2%, *p* = 0.011). In general, rates did not vary by surgeon age, practice setting nor geographic location; however, those who had been in practice for >30 years were also significantly less likely to state that they would “*always*” order LFTs than those who had been in practice for <5 years (42.6 vs. 72.0%, *p* = 0.027).

## Discussion

Every year, over 232,000 women will be diagnosed with breast cancer; the majority of these at early stage (1). It is estimated that the number of patients diagnosed with breast cancer will increase significantly by 2030 and, therefore, excess expenditure without added benefit in these patients is worthy of consideration. We found that there was significant variation among surgeons in the preoperative workup of relatively straight-forward early breast cancer patients.

### Additional Breast Imaging

In terms of additional imaging, there seemed to be an equal distribution of respondents who stated that they would either “*never*,” “*rarely*,” “*sometimes*,” “*often*,” or “*always*” order a breast ultrasound. NCCN guidelines recommend that breast ultrasound be used “*as necessary*” as an adjunct to mammography in the workup of patients with early stage breast cancer (2). The term “*as necessary*” is not highly prescriptive, and may explain wide variations in practice. Some authors have argued that the addition of whole-breast ultrasound may find clinically and mammographically occult cancers that may impact the patient’s care, particularly when multicentric (3–5), and may also improve margin clearance (6). However, the utility and cost-effectiveness of routine use of whole-breast ultrasound has not been established. It is likely that breast ultrasound may be useful in some circumstances but not in all. Still, we found that nearly a quarter of surgeons who responded to the survey stated that they would *always* order a breast ultrasound, and those who were in solely breast-related practices were more likely to claim that they do so. This raises the issue of whether breast ultrasound is over-utilized in these practices, and one may speculate that breast surgeons with ready access to ultrasonography may be more likely to either order or perform breast ultrasound because of its immediate availability rather than because the outcome of the test would truly change their management.

Similarly, the utility of MRI as a routine preoperative measure has been questioned. While it is clear that MRI may find additional disease that may be otherwise mammographically occult, it is unclear whether such disease would be clinically significant. For example, it is well-known that MRI can find multicentric disease in up to 30% of patients (7). Yet, clinical trials like the NSABP B-06, which were done in the pre-MRI era, found no difference in survival between those undergoing partial vs. total mastectomy, leading some to question the impact of undetected multicentric disease on this outcome (8). Indeed, an individual level meta-analysis found that MRI had no impact on overall or disease-free survival (9). While some have argued that preoperative MRI may allow better evaluation of extent of disease, thereby yielding better margin clearance, there have been two randomized controlled trials that have produced level 1 evidence to the contrary (10, 11). While the majority of surgeons in our study stated that they would “*never*” or “*infrequently*” use preoperative breast MRI in the situation of an asymptomatic patient presenting with a mammographically detected, non-palpable invasive ductal carcinoma, 4.1% of surgeons stated that they “*always*” would despite clear evidence of the lack of utility for this modality as standard routine practice (12).

Interestingly, we found a relationship between the propensity to order a preoperative MRI and that to order breast ultrasound, such that 28.6% of patients who stated that they would “*always*” order a breast MRI would also “*always*” order a breast ultrasound. It is unclear whether surgeons who responded in this way would “*always*” order an ultrasound to further evaluate findings on breast MRI. If this was the case, however, this would assume that they always found additional lesions found on MRI warranting further ultrasonographic evaluation, or that they routinely ordered ultrasound upfront “just in case” the MRI found such lesions. Perhaps more likely, these respondents would “*always*” order an MRI to further evaluate patients after breast mammogram and ultrasound. However, Mariscotti et al. found minimal incremental benefit of adding MRI to patients evaluated with mammography and ultrasound (13).

In a study of women diagnosed with breast cancer in the SEER-Medicare database, Lee et al. found that 28.5% of patients would have additional breast imaging studies (beyond mammography). These data are similar to our findings. While we did not specifically evaluate costs, as these would be different at different institutions around the country and the globe, the effect of additional breast imaging on the cost of care is not insignificant. Lee et al. noted that “the average *per capita* cost for a diagnostic workup was $360.89 in 2004, about half of which was attributable to relevant radiology, diagnostic mammography, and other breast imaging studies” (14). Given the ubiquity of breast cancer, these data highlight the need to carefully evaluate variation in the use of breast imaging studies, and the utility and the cost-effectiveness of such practices, particularly given our findings that surgeons with a greater proportion of breast-related practices were more likely to order further breast imaging.

### Metastatic Workup

The majority of surgeons surveyed followed ASCO/NCCN guidelines (2, 15) and stated that they would “*never*” order a metastatic workup in asymptomatic patients with mammographically detected, non-palpable breast cancers. However, the proportion of surgeons who stated that they would “never” order tests that would be considered part of a metastatic workup ranged from 56% (for CT chest) to 71% (for either ultrasound of the liver or CT/MRI of the brain). This leaves a significant minority of surgeons who would at least infrequently order these tests in patients who were otherwise asymptomatic with a non-palpable mammographically detected lesion, which would presumably be early stage disease. Our finding that up to 5% of respondents would “*always*” order tests, such as CT scans or bone scans, in patients with presumably early stage disease is concerning, particularly given that the cost of detecting metastatic disease per patient screened in this population is nearly infinite (16).

We have previously reported the data in which we found that 7.3% of surgeons “always” performed a metastatic workup prior to surgery (17), even though the probability of finding metastatic disease in patients with early stage breast cancer is <1–2% (18). Not only is avoiding a metastatic workup in asymptomatic patients who present with early stage disease a recommendation of ASCO’s “Choosing Wisely” campaign and the NCCN guidelines, but also such a policy is endorsed internationally. For example, the European guidelines for quality assurance in breast cancer screening and diagnosis stated that “a complete diagnostic work-up to detect metastases is unnecessary in the majority of patients with newly diagnosed breast cancer whereas it may be indicated for patients with advanced disease” (19). The European Society for Medical Oncology also noted that, aside from physical examination, “other tests are not routinely recommended unless locally advanced or when symptoms suggestive of metastases are present” (20). Practice guidelines from Canada similarly state that “routine bone scanning, liver ultrasonography and chest radiography are not indicated before surgery” (21).

### Chest X-Rays and “Routine” Blood work

Over a third of surgeons stated that they would “*always*” order a CXR as part of their preoperative workup in a patient with early breast cancer. It is unclear as to whether this was done to rule out metastatic disease or whether it was done to stratify patients’ operative risk. From an oncologic standpoint, NCCN guidelines do not recommend CXR in this setting (2). Furthermore, the American Society of Anesthesiologists and other professional organizations do not recommend routine CXR as part of the perioperative workup of asymptomatic patients (22, 23).

While NCCN guidelines continue to recommend routine blood work, including a CBC and liver function studies, the true value of these studies in terms of detecting conditions or metastatic disease that would change management is unclear. Nonetheless, the majority of surgeons surveyed stated that they would “*always*” perform these tests in accordance with guidelines. Of note, the NCCN guidelines do *not* recommend CMP/electrolytes; yet, 53.3% of surgeons stated that they “*always*” ordered these tests. Some may argue that these tests are being ordered as part of a perioperative workup, rather than as a result of the breast cancer diagnosis. However, the American Society of Anesthesiologists recommends against routine preoperative testing (22); rather “preoperative electrolyte and creatinine testing should be reserved for patients at risk of electrolyte abnormalities or renal impairment” (24).

Studies from around the world have found an over-utilization of “routine” blood work in the perioperative setting without a concomitant benefit in terms of changing management (25–27). Issa et al. found that of the 1075 preoperative tests done in 200 patients in Brazil, 55.8% were not indicated (27). Interestingly, they found that surgeons, more than anesthesiologists, order tests as a matter of routine resulting in a net increase in the cost per patient of 33.6% (27). Similarly, in a study of 1496 patients in Thailand, Siriussawakul et al. found that only 12.1% of patients had preoperative workups that were in accordance with guidelines, with CMP/electrolytes being the most commonly ordered unnecessary test (26). In the UK, Phoenix et al. estimated that inappropriate preoperative blood work resulted in an extrapolated cost of £11.2 million per year (28). In the United States, a similar trend is noted. St. Clair et al. found that only 17% of preoperative CMPs were indicated. The cost of the unnecessary CMP in the 197 patients in whom it was ordered without indication in their study was estimated to be $9,589 based on Medicare reimbursement and $41,670 based on institutional charges (25).

### Study Limitations

This study should be considered in the context of its limitations. To begin with, this is a survey of surgeons. As such, it is limited by the subjective responses of surgeons. We had no way of objectively validating surgeons’ responses, nor could we assess the outcomes of variations in practice pattern for individual patients. However, this survey asked surgeons to describe their usual practice, and how often they would order tests in an otherwise healthy asymptomatic patient presenting with a non-palpable cancer found as a result of a mammographic abnormality. Second, the surgeons who responded to this survey were self-selected, and we had no way of determining a response rate, nor understanding whether the cohort of surgeons who responded to the survey were systematically different from those who did not. Having said this, the demographics of the surgeons who responded are diverse and seem to reflect, in general, the practice of breast surgery in the United States. Furthermore, we did not query surgeons who stated that they would “*always*” order studies that are not recommended as a matter of guidelines as to whether they (a) knew that their practice was not indicated and (b) why they continued to advocate for such tests. It is possible that surgeons order such tests due to patient insistence, medical malpractice concerns, or institutional practices that do not comply with guidelines.

## Conclusion

Despite these limitations, this study highlights variation in practice in the workup of early stage breast cancer patients. As healthcare costs continue to rise at an unsustainable pace, there is increasing focus on evidence-based cost-effective care. Our study, which found wide variation in the diagnostic and preoperative workup of breast cancer patients, is particularly concerning given the prevalence of this disease. Despite guidelines, surgeons will often order tests that may not be needed, highlighting the need for ongoing education, and the potential for tremendous cost-savings if variation could be minimized. This study should call attention to the over-utilization of tests that add minimal incremental benefit. Professional organizations and institutions should re-evaluate their current policies and engage in further education of surgeons to “choose wisely” when ordering workups for their otherwise healthy newly diagnosed asymptomatic breast cancer patients.

## Author Contributions

All authors contributed to the design of the study, collection of data, interpretation of findings, and all have reviewed and approved the final manuscript. This work is being done and submitted on behalf of the American College of Surgeons Communities.

## Author Note

Presented at the World Surgical Congress, Bangkok, Thailand, August 25, 2015.

## Conflict of Interest Statement

The authors declare that the research was conducted in the absence of any commercial or financial relationships that could be construed as a potential conflict of interest.
